# Thulium Fibre Laser Versus Pulse-Modulated Holmium:Yttrium Aluminum Garnet (Ho:YAG) Laser for Endoscopic Lithotripsy: A Systematic Review of Clinical Outcomes, Efficiency, and Safety

**DOI:** 10.7759/cureus.97030

**Published:** 2025-11-17

**Authors:** Maanya Bhardwaj, Abhinav Singhal, Gaurika Bhardwaj, Layla Sultan, Maitrey Darrad

**Affiliations:** 1 Urology, Cambridge University Hospital NHS Foundation Trust, Cambridge, GBR; 2 Urology, University Hospitals Birmingham NHS Foundation Trust, Birmingham, GBR; 3 Surgery, Royal Brompton and Harefield NHS Foundation Trust, London, GBR; 4 Surgery, University Hospitals Birmingham NHS Foundation Trust, Birmingham, GBR; 5 Urology, University Hospitals Birmingham NHS Foundation Trust, Birmimgham, GBR

**Keywords:** endourology, holmium:yag laser (ho:yag), laser lithotripsy, operative time, pulse modulation, retrograde intrarenal surgery (rirs), stone-free rate (sfr), thulium fiber laser (tfl), ureteroscopy (urs), urolithiasis

## Abstract

The Holmium:Yttrium Aluminum Garnet (Ho:YAG) laser has been the standard energy source for endoscopic lithotripsy for over two decades; however, limitations such as inefficient energy transmission, strong retropulsion, and large fiber diameters have prompted the development of newer laser technologies. Pulse-modulated Ho:YAG platforms (e.g., Moses™ (Boston Scientific, Marlborough, USA), Virtual Basket™ (Quanta System, Milan, Italy), Magneto™ (Quanta System, Milan, Italy)) and the Thulium Fibre Laser (TFL) were introduced to improve energy coupling, fragmentation efficiency, and surgical precision. This systematic review aims to evaluate and compare the clinical efficacy and safety of TFL and pulse-modulated Ho:YAG systems in the management of urolithiasis.

A systematic search of PubMed, Cochrane Library, and Embase was conducted following the Preferred Reporting Items for Systematic Reviews and Meta-Analyses (PRISMA) guidelines. Studies comparing TFL and pulse-modulated Ho:YAG lasers in ureteroscopy (URS), retrograde intrarenal surgery (RIRS), or mini-percutaneous nephrolithotomy (mini-PCNL) were included. Data on stone-free rate (SFR), operative and lasering times, retropulsion, visibility, complications, and safety were extracted. Due to heterogeneity in definitions and study design, a qualitative synthesis without meta-analysis (SWiM) was performed. Risk of bias was assessed using the Risk of Bias (RoB) 2.0 (The Cochrane Collaboration, London, UK) and ROBINS-I (The Cochrane Collaboration, London, UK), and evidence certainty was graded with Grading of Recommendations Assessment, Development and Evaluation (GRADE).

Eight studies, including two randomized controlled trials and six observational studies, were included in the review. Across most studies, TFL achieved similar or slightly higher SFRs (82-98%) compared with pulse-modulated Ho:YAG (56-95%). Operative and lasering times were comparable, while TFL demonstrated less retropulsion, superior endoscopic visibility, and an equivalent complication profile. No clinically significant differences in thermal safety or re-hospitalization were observed.

In summary, TFL provides comparable or marginally superior performance to pulse-modulated Ho:YAG lasers for endoscopic stone surgery. It offers consistent safety and improved intraoperative control, suggesting an emerging role as a preferred next-generation lithotripsy platform pending further multicenter randomized trials.

## Introduction and background

Urolithiasis or Kidney stone disease (KSD) remains a major global health issue, affecting approximately 10-15% of the world's population, with recurrence rates approaching 50% within 10 years [1]. Advances in endourological technology have made ureteroscopy and laser lithotripsy the mainstay of treatment for most upper urinary tract stones [2].

Conventional Holmium:Yttrium Aluminum Garnet (Ho:YAG) lasers have long been the gold standard for lithotripsy, but are limited by inefficient energy transfer, significant stone retropulsion, large fibre diameters, and restricted pulse frequency, which subsequently reduces ablation efficiency and control during endoscopic procedures [3,4,5]. To overcome these drawbacks, newer laser technologies have recently emerged, promising to improve fragmentation efficiency and procedural ergonomics. Two major innovations include: (a) the Thulium Fibre Laser (TFL), operating at approximately 1940 nm with higher water absorption and smaller fibre diameters; and (b) pulse-modulated Ho:YAG systems, such as Moses™ (Boston Scientific, Marlborough, USA), Virtual Basket™ (Quanta System, Milan, Italy), Magneto™ (Quanta System, Milan, Italy)​​​​​​**, **which reshape the laser pulse to improve energy transmission and reduce stone retropulsion [6,7]. 

Thulium Fibre Laser has been shown to produce faster ablation, finer dusting, and less retropulsion compared with conventional Ho:YAG [8,9,10]. These benefits are attributed to its higher water absorption coefficient, more stable energy delivery, and higher pulse frequency capability [8,9]. Moreover, TFL fibres are thinner, allowing improved irrigation and greater flexibility during retrograde intrarenal surgery (RIRS) and ureteroscopic (URS) procedures [9]. 

Despite these promising technical advantages, it remains unclear whether TFL offers superior clinical outcomes when compared with the latest pulse-modulated Ho:YAG systems. Most available systematic reviews and meta-analyses have grouped conventional Ho:YAG and pulse-modulated Ho:YAG systems together, thereby limiting the ability to draw specific conclusions about the latest pulse-modulated technologies [6,11]. This systematic review aims to compare the Thulium Fiber Laser and pulse-modulated Ho:YAG lasers in the management of renal stones. 

## Review

Methods

This systematic review was conducted in accordance with the Preferred Reporting Items for Systematic Reviews and Meta-Analyses (PRISMA) guidelines [12]. A comprehensive search was conducted in the following electronic databases: PubMed, MEDLINE, PMC, Cochrane Library, and Embase. Articles published from 1st January 2017 (reflecting the introduction of pulse-modulated Ho:YAG systems) to 1st October 2025 were included in the search. Studies were selected according to the inclusion and exclusion criteria specified in Table 1. 

**Table 1:** Inclusion and exclusion criteria for the studies RCT: Randomized Controlled Trial, TFL: Thulium Fibre Laser, Ho:YAG: Holmium:Yttrium Aluminum Garnet, RIRS: Retrograde Intrarenal Surgery, URS: Ureteroscopy, mini-PCNL: Miniaturized Percutaneous Nephrolithotomy, KTP: Potassium Titanyl Phosphate, SFR: Stone-Free Rate, BPH: Benign Prostatic Hyperplasia

Category	Inclusion Criteria	Exclusion Criteria
Study Design	RCTs, prospective, or retrospective comparative cohort studies, case–control studies	Non-comparative studies (TFL or Ho:YAG alone), case reports, case series, reviews, editorials, expert opinions, preclinical, animal, or in-vitro (bench) studies
Population	Adult patients (≥18 years) with urolithiasis (renal, ureteric, or bladder stones), patients undergoing endoscopic laser lithotripsy (RIRS/URS/mini-PCNL)	Paediatric populations, non-urolithiasis indications (e.g., BPH, strictures), mixed data where urolithiasis outcomes cannot be separated
Intervention	Lithotripsy using TFL of any power setting or mode	Not applicable
Comparator	Lithotripsy using pulse-modulated Ho:YAG laser, including Moses™, Virtual Basket™, Magneto™, or equivalent technologies	Conventional (non-modulated) Ho:YAG lasers, other laser types (e.g., KTP, diode)
Outcomes	At least one of the following outcomes reported: stone-free rate (SFR), operative time, laser activation time, retropulsion or fragment migration, intraoperative complications, postoperative complications, readmission or re-intervention rate	Studies lacking quantitative clinical outcomes, studies focused only on laser physics or ablation efficiency (no patient outcomes)
Language	English	Studies not written in English
Publication Date	January 2017 to October 2025 (reflecting introduction of pulse-modulated Ho:YAG systems)	Studies not conducted in this time frame

**Figure 1:**
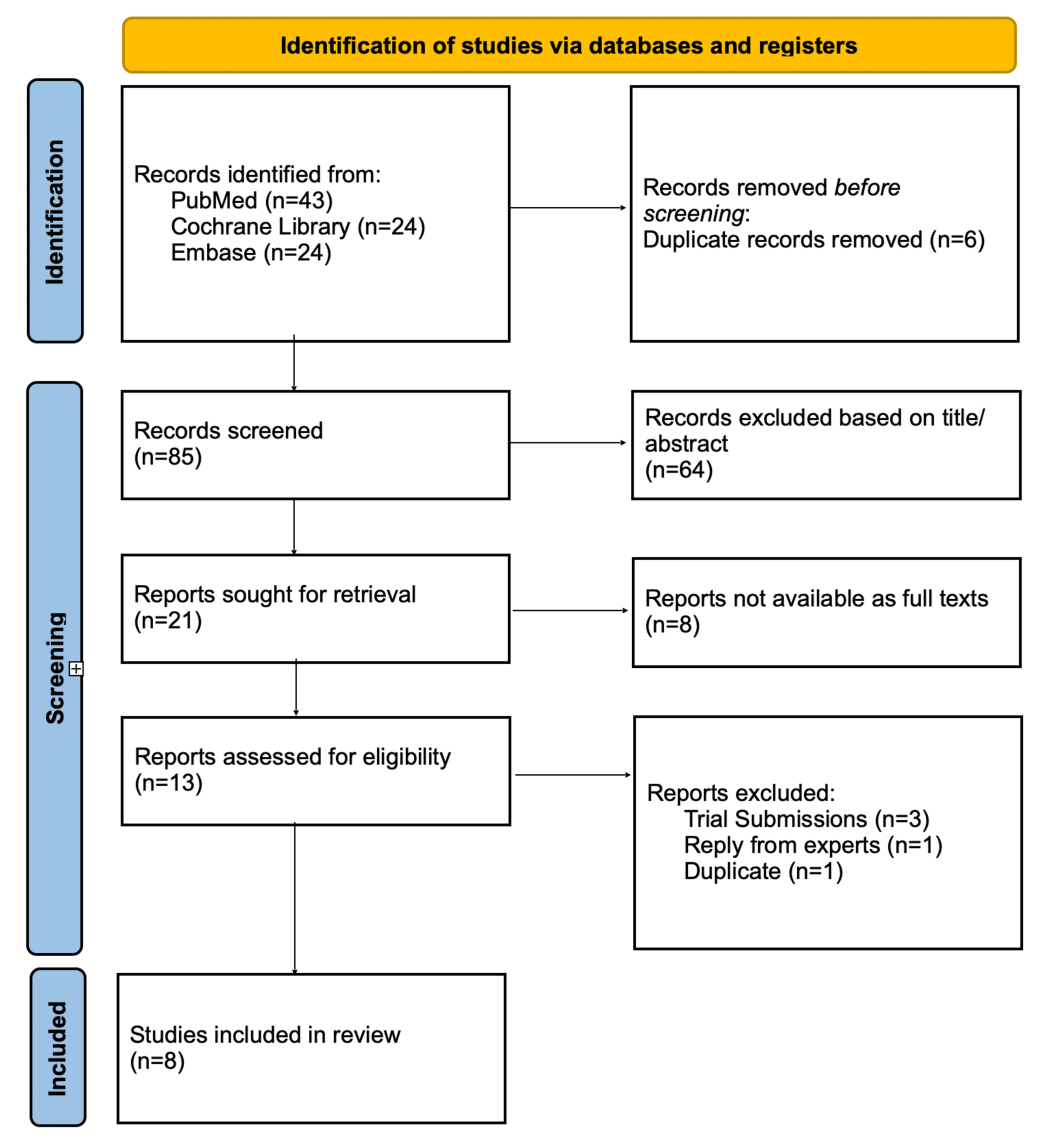
PRISMA flow diagram for selecting studies for inclusion in the review PRISMA: Preferred Reporting Items for Systematic Reviews and Meta-Analyses

A structured search strategy was developed using keywords and Medical Subject Headings (MeSH) terms. The search terms included combinations of Thulium Fibre Laser (TFL), Holmium:YAG Laser (Ho:YAG), Pulse Modulation, Laser Lithotripsy, Urolithiasis, Ureteroscopy (URS), Retrograde Intrarenal Surgery (RIRS), Stone-Free Rate (SFR), Operative Time, and Endourology. Search strategies used to identify relevant papers from various databases are available in the Appendices. Titles and abstracts were screened independently by three reviewers (AS, MB, and GB) using the inclusion and exclusion criteria specified in Table 1. Full texts for potentially eligible studies were retrieved and evaluated for final inclusion. The PRISMA flow diagram in Figure 1 provides an overview of the process used for selecting studies for the review [12]. Data was extracted independently by two reviewers (AS and MB) using a standardized data extraction form. The data collected included study characteristics, population details, intervention details, outcome measures, key findings, and conclusions. Disagreements between reviewers were resolved through discussion and re-examination of the eligibility criteria. Persistent discrepancies were adjudicated by a third reviewer (GB) to ensure consensus. 

A quantitative meta-analysis was not undertaken due to substantial heterogeneity across the included studies. The identified reports varied considerably in laser technology, procedural settings, and study design, with the majority of the studies being small, single-center observational studies. Definitions of key outcomes, such as stone-free rate and operative efficiency, were inconsistent, employing different imaging modalities and follow-up intervals. Given these methodological and reporting differences, statistical pooling was deemed inappropriate [13]. 

Therefore, a structured qualitative synthesis was performed in accordance with PRISMA 2020 and synthesis without meta-analysis (SWiM) guidelines [12,14]. The direction and consistency of effects were summarized narratively, with greater weight given to higher-quality or randomized studies, and risk-of-bias assessments were incorporated into the interpretation of results [15]. The methodological quality of included studies was assessed using the Cochrane Risk of Bias (RoB) 2.0 [16] tool for randomized controlled trials and the Risk Of Bias In Non-randomized Studies - of Interventions (ROBINS-I) tool [17] for non-randomized studies. Each domain was rated as low, moderate, or high risk of bias. 

Results

Eight studies comparing TFL and pulse-modulated Ho:YAG systems met the inclusion criteria. This included a total of 2,404 patients across RIRS, URS, and miniaturized percutaneous nephrolithotomy (mini-PCNL) procedures between 2022-2025 (Table 2). Study designs comprised two randomized controlled trials (RCTs) [18,19] and six comparative observational studies [20,21,22,23,24,25]. Sample sizes ranged from 66 to 1,567. All Ho:YAG comparators utilized pulse-modulated platforms such as MOSES™ 1.0/2.0, Virtual Basket™, or Magneto™ technologies. TFL sources were predominantly SuperPulsed™ systems (SOLTIVE™ or equivalent (Olympus Corporation, Tokyo, Japan)).

**Table 2:** Key characteristics of included studies RCT: Randomized Controlled Trial, URS: Ureteroscopy, RIRS: Retrograde Intrarenal Surgery, TFL: Thulium Fibre Laser, Ho:YAG: Holmium:Yttrium Aluminium Garnet, SFR: Stone-Free Rate, CT: Computed Tomography, PSM: Propensity Score Matched, Mini-PCNL: Miniaturized percutaneous nephrolithotomy SuperPulsed™ TFL (SOLTIVE™) by Olympus Corporation, Tokyo, Japan

Author (Year)	Design	Procedure	Laser Type (Comparators)	Sample Size (TFL / Ho:YAG)	Key Outcomes	Main Findings
Haas et al. 2023 [18]	RCT, single-center	URS/RIRS (renal + ureteric stones < 2 cm)	SuperPulsed™ TFL (SOLTIVE™) vs Pulse-Modulated Ho:YAG (Moses 2.0)	56 / 52	URS time, SFR, complications	No significant difference in URS time (19.9 vs 21.4 min), SFR, or complications
Gupta et al. 2025 [19]	RCT, single-center	RIRS (renal stones 5–20 mm)	SuperPulse TFL (Olympus Soltive) vs Pulse-Modulated Ho:YAG (Moses 2.0)	33 / 33	SFR (CT 6 weeks), laser efficiency, complications	SFR 82 % (TFL) vs 79 % (Ho:YAG); no difference in efficiency or safety
Castellani et al. 2023 [20]	Multicenter registry (PSM)	RIRS (renal stones)	TFL (varying brands) vs Ho:YAG (Moses)	1567 / 508 (raw) → 284 / 284 (PSM)	SFR, complications	TFL higher SFR (85% vs 56%, p<0.001); sepsis higher with TFL (3.2 % vs 0 %)
Geavlete et al. 2022 [21]	Retrospective, single-center	RIRS (± second-look URS)	TFL (Soltive) vs Ho:YAG (Moses)	59 / 187	True SFR at 3 months	SFR 96.6 % (TFL) vs 86.6 % (Ho:YAG); fewer residuals with TFL
Vergamini et al. 2024 [22]	Retrospective comparative	Mini-PCNL	SuperPulsed TFL vs Ho:YAG (Moses)	50 / 50	Operative time, SFR, efficiency	Ho:YAG shorter lasering and operative times; SFR similar
Rico et al. 2025 [23]	Retrospective comparative	RIRS with suction sheath	TFL vs Magneto Ho:YAG	66 / 61	SFR, operative time, efficiency	SFR 98 % (TFL) vs 94.7% (Ho:YAG); no significant differences
Kudo et al. 2025 [24]	Retrospective comparative (single-center)	RIRS for renal and ureteric stones	Thulium Fiber Laser (Olympus Soltive) vs Ho:YAG with Moses technology	46 / 48	SFR, operative time, complications, visibility score	SFR TFL 91 % vs Ho:YAG 87% (NS); operative time shorter for TFL (47 ± 12 vs 54 ± 13 min, p<0.05); better visibility and lower retropulsion with TFL; complication rates similar
Yang et al. 2024 [25]	Prospective cohort	Flexible URS	TFL vs Pulse-modulated Ho:YAG	90 / 90	SFR, operative parameters	SFR 92% (TFL) vs 85% (Ho:YAG); less retropulsion and fiber degradation with TFL

Primary Outcomes

Stone-Free Rate (SFR): All studies assessed SFR using either CT Kidneys, Ureters, Bladder (KUB), or intraoperative endoscopic confirmation at 2-12 weeks. SFRs ranged from 79-98% for TFL and 56-95% for pulse-modulated Ho:YAG. Four studies [20, 21, 25, 24] reported numerically higher SFRs with TFL, while three studies [18, 19, 23] showed no statistical difference. No study demonstrated significant superiority of Ho:YAG in SFR. 

Operative Time: Five studies provided operative duration, with three studies [18, 19, 23] showing no significant difference. Kudo et al [24] demonstrated shorter operative time with TFL (47 ± 12 vs 54 ± 13 min, p < 0.05), while Vergamini [22] (mini-PCNL) favoured pulse-modulated Ho:YAG for efficiency (shorter lasering and operative time). Overall, the trend suggests comparable or slightly shorter operative times with TFL in flexible ureteroscopic procedures. 

Secondary Outcomes

Four studies [23, 25, 21, 18] evaluated retropulsion, with TFL demonstrating significantly less stone retropulsion and improved endoscopic visibility in two [24, 25]. The remaining studies reported comparable retropulsion behavior between systems. Across all studies, overall complication rates were low (2-10%), mainly Clavien-Dindo grade I-II events such as transient hematuria or fever. No major laser-related ureteric injuries or perforations were observed. Only Castellani [20] reported a higher sepsis rate in the TFL group (3.2% vs 0% Ho:YAG), though this was not clinically significant. 

Both laser systems maintained intra-renal temperatures within safe limits (<43 °C), with Kudo et al. [24] and Rico et al. [23] noting no evidence of thermal injury. Hospital stay and re-intervention rates were largely similar across studies, and cost comparisons were limited, though TFL was noted to have lower fiber degradation and longer fiber lifespan (Yang et al. [25]). Subjective assessments from Kudo et al. [24] and Haas et al. [18] indicated that TFL offered smoother dusting, reduced retropulsion, and enhanced visibility, contributing to greater overall surgeon satisfaction. 

Risk of Bias Assessment

Overall study quality was acceptable, with two randomized controlled trials [18, 19] and six comparative observational studies. Both RCTs clearly described randomization and predefined outcomes; Haas et al. [18] demonstrated low risk across all domains, while Gupta et al. [19] showed minor concerns regarding allocation concealment and blinding. Outcome measurement and completeness were appropriate in both, and no selective reporting was detected. 

Among the non-randomized studies [20-25], risk of bias was generally moderate. Selection and confounding biases were common due to retrospective or single-center designs, although propensity matching [20] and prospective data collection [25] mitigated some limitations. Outcome assessment bias was low because endpoints such as SFR and operative time were objectively defined, though imaging and follow-up protocols varied. No study showed major attrition or selective outcome reporting. Overall, evidence certainty is moderate, supporting qualitative synthesis rather than statistical pooling. Table 3 summarizes the risk of bias assessment for the included studies.

**Table 3:** Summary of Risk of Bias assessment RCT: Randomized Controlled Trials, RoB 2: Cochrane Risk of Bias 2 tool (The Cochrane Collaboration, London, UK), ROBINS-I: Risk Of Bias In Non-randomized Studies-of Interventions (The Cochrane Collaboration, London, UK)

Study	Design	RoB Tool	Bias in Randomization/Confounding	Bias in Measurement	Incomplete Data	Selective Reporting	Overall Risk
Haas et al. 2023 [18]	RCT	RoB 2	Low	Low	Low	Low	Low
Gupta et al. 2025 [19]	RCT	RoB 2	Some concerns (small sample, unclear concealment)	Low	Low	Low	Some concerns
Castellani et al. 2023 [20]	Registry (PSM)	ROBINS-I	Moderate (selection bias)	Moderate	Low	Low	Moderate
Geavlete et al. 2022 [21]	Retrospective	ROBINS-I	Serious (selection, unbalanced baseline)	Low	Low	Moderate	Serious
Vergamini et al. 2024 [22]	Retrospective	ROBINS-I	Moderate	Low	Low	Low	Moderate
Rico et al. 2025 [23]	Retrospective	ROBINS-I	Moderate	Low	Low	Low	Moderate
Kudo et al. 2025 [24]	Retrospective	ROBINS-I	Moderate	Low	Low	Low	Moderate
Yang et al. 2024 [25]	Prospective cohort	ROBINS-I	Low–moderate	Low	Low	Low	Low–moderate

Certainty of Evidence 

The certainty of evidence for SFR was moderate, supported by two randomized trials and observational data showing that TFL yields similar or slightly higher SFR than pulse-modulated Ho:YAG. Operative and lasering times were rated at low certainty due to inconsistency across procedures (URS/RIRS/mini-PCNL) and imprecision in reporting. Evidence for retropulsion/visibility and complications reached moderate certainty, indicating less retropulsion with TFL and no difference in adverse events overall. Thermal safety, hospitalization/re-intervention, and cost/satisfaction remained low to very-low certainty given sparse, heterogeneous, or surrogate outcomes. Collectively, these ratings support a conclusion that TFL is at least non-inferior, with possible incremental advantages, compared with pulse-modulated Ho:YAG in contemporary endoscopic lithotripsy. Table 4 summarizes the Grading of Recommendations Assessment, Development, and Evaluation (GRADE)assessment for the studies included. 

**Table 4:** GRADE assessment for included studies SFR: Stone-Free Rate, RCT: Randomized Controlled Trial, Ho:YAG: Holmium:Yttrium Aluminium Garnet, TFL: Thulium Fibre Laser, Mini-PCNL: Miniaturized Percutaneous Nephrolithotomy, obs: Observational Study, ↓: Downgraded, GRADE: Grading of Recommendations Assessment, Development, and Evaluation

Outcome	Studies (design)	Overall direction of effect	GRADE certainty	Key reasons for rating (downgrades/notes)
Stone-free rate (SFR)	8 (2 RCTs + 6 obs.)	Favours TFL or no difference (no study favoured Ho:YAG)	Moderate	↓ Risk of bias (non-randomized dominance), ↓ Inconsistency (mixed SFR definitions/imaging). Two low-risk RCTs anchor certainty upward.
Operative time	5 (2 RCTs + 3 obs.)	Mostly no difference; one study favoured TFL; one (mini-PCNL) favoured Ho:YAG	Low	↓ Inconsistency (procedure setting, surgeon/center effects), ↓ Imprecision (small trials; variable reporting).
Lasering time	3 (1 RCT + 2 obs.)	No consistent difference	Low	↓ Imprecision (few studies, small N), ↓ Inconsistency (settings/metrics differ).
Retropulsion/visibility	4 (1 RCT + 3 obs.)	Favours TFL (less retropulsion; better visibility)	Moderate	↓ Risk of bias (subjective scales; non-randomized), but consistent direction across settings.
Complications	7 (2 RCTs + 5 obs.)	No difference (low overall rates)	Moderate	↓ Risk of bias (observational designs); events infrequent. One registry noted higher sepsis with TFL without downstream sequelae.
Thermal safety	2 (obs.)	No clinically relevant thermal injury with either laser	Low	↓ Indirectness (surrogate measures; heterogeneous monitoring), ↓ Imprecision (limited datasets).
Hospitalization/re-intervention	4 (obs.)	No difference	Low	↓ Risk of bias, ↓ Imprecision (few events; variable follow-up).
Cost/surgeon satisfaction	2–3 (obs.)	Insufficient; trends favour TFL for visibility/handling	Very low	↓↓ Serious imprecision (sparse, subjective), ↓ Indirectness (non-standardized instruments), ↓ risk of bias.

Discussion

This systematic review consolidates contemporary clinical evidence comparing TFL and pulse-modulated Holmium:YAG (Ho:YAG) systems in endoscopic stone surgery. Across eight studies published between 2022 and 2025, TFL achieved equivalent or superior clinical outcomes in SFR, operative efficiency, and safety. Although the heterogeneity of study designs precluded meta-analysis, qualitative synthesis demonstrated a consistent direction of benefit toward TFL in several operative domains. 

Clinical Outcomes and Operative Performance 

The pooled qualitative findings suggest that TFL achieves comparable or slightly higher SFRs relative to pulse-modulated Ho:YAG systems such as Moses™, Virtual Basket™, and Magneto™. Several studies [20, 21, 24, 25] demonstrated a numerical improvement in SFR, particularly for renal calculi managed by RIRS. This may be attributable to the higher pulse frequency, lower pulse energy, and smaller fibre diameter characteristic of TFL, which promote finer fragmentation and reduced retropulsion. In contrast, trials by Haas et al. [18] and Gupta et al. [19] found no significant difference, reinforcing that both technologies remain clinically effective and safe when used optimally. 

Operative and lasing times were largely equivalent, although Kudo et al. [24] reported shorter operative durations with TFL, and Vergamini et al. [22] found greater energy efficiency with Ho:YAG in mini-PCNL. These discrepancies likely reflect procedural heterogeneity, surgeon experience, and differing laser settings rather than intrinsic limitations of either technology. Importantly, no study identified increased complication rates or adverse thermal events with TFL, confirming its favourable safety profile even at high-power, high-frequency settings. 

*Mechanistic and Technical Considerations* 

The physical differences between laser sources may explain the subtle clinical advantages of TFL. The 1940-nm wavelength of TFL is better absorbed in water, enabling more controlled vapour bubble formation and continuous “fine dusting” ablation. This, combined with low pulse energy and high repetition rates (up to 2000 Hz), minimizes stone retropulsion and improves endoscopic visibility [3,4]. Conversely, pulse-modulated Ho:YAG systems achieve comparable dusting through energy delivery modulation (e.g., Moses effect), but their shorter wavelength and lower absorption coefficient can result in more turbulent irrigation and reduced control in confined calyces [4,5]. The qualitative evidence herein suggests these physical advantages translate to incremental improvements in efficiency and visibility, though the magnitude of benefit remains modest. 

Complications and Safety 

Across all included studies, complication rates were low (2-10%) and predominantly Clavien-Dindo grade I-II. Only one study [20] reported a higher postoperative sepsis rate in the TFL group, but without significant clinical sequelae. No ureteral perforations or thermal injuries were recorded. Measurements of intrarenal temperature in studies such as Kudo et al. [24] confirmed values remained well below the 43 °Csafety threshold. These findings suggest that both laser systems are safe and clinically reliable, provided irrigation and energy settings are appropriate. 

Interpretation in the Context of Existing Literature 

Previous systematic reviews comparing TFL and conventional Ho:YAG lasers [6, 7, 10] also found equivalent or slightly superior outcomes for TFL. The present synthesis refines these conclusions by focusing specifically on pulse-modulated Ho:YAG systems, which were designed to close the performance gap with TFL. Even against these advanced comparators, TFL maintained similar or improved outcomes, particularly regarding SFR, retropulsion, and surgeon-rated visibility. These observations support the notion that pulse modulation narrows, but does not eliminate, the inherent physical advantages of TFL. 

Risk of Bias and Evidence Certainty 

The overall methodological quality of the included studies was moderate. The two RCTs [18, 19] were low-risk, while most observational studies were subject to confounding and selection bias inherent to their design. Definitions of key outcomes, such as SFR and efficiency, varied, and imaging modalities were not standardized. Nevertheless, outcome measures were objective, attrition was minimal, and reporting was transparent. Consequently, the certainty of evidence supporting TFL’s non-inferiority and possible marginal superiority is moderate and suitable for qualitative synthesis. 

Limitations 

There are several limitations, such as heterogeneity in laser settings, fibre diameters, and procedural contexts (URS vs RIRS vs mini-PCNL), which limited quantitative comparison. Secondly, follow-up intervals and imaging modalities for SFR were inconsistent, ranging from intraoperative assessment to CT KUB at 12 weeks post-procedure. Most data were from single-center or manufacturer-associated studies, which may influence generalizability. Finally, cost analysis and long-term outcomes, such as fibre durability and re-treatment rates, remain underreported. 

Clinical and Research Implications

From a clinical standpoint, both TFL and pulse-modulated Ho:YAG systems represent safe and effective laser platforms for endoscopic lithotripsy. TFL may offer practical advantages, including lower retropulsion, finer dusting, and enhanced visibility, that could improve workflow efficiency and reduce the need for basket extraction. For centers already equipped with pulse-modulated Ho:YAG systems, upgrading to TFL should be guided by case complexity, budget, and procedural volume rather than expectation of dramatic clinical superiority. Future research should prioritize large multicenter randomized trials directly comparing high-power TFL and Moses 2.0 systems under standardized protocols, with consistent definitions of SFR, energy efficiency, and thermal safety. 

## Conclusions

Thulium Fibre Laser demonstrates equivalent or marginally superior clinical outcomes compared with pulse-modulated Holmium:YAG systems for endoscopic management of urolithiasis. Across contemporary studies, TFL achieved similar or higher stone-free rates, comparable operative efficiency, and a consistently low complication profile, with advantages in retropulsion control and endoscopic visibility. Both platforms remain safe and effective for ureteroscopic and intrarenal lithotripsy, but TFL may offer incremental technical benefits that enhance procedural precision. Further large, multicenter randomized trials are warranted to confirm these findings and establish standardized metrics for laser performance and efficiency. 

## Appendices

Appendix A

**Table 5:** Search strategy for the databases

Search strategy	Database Used	Number of papers filtered
(("thulium fiber laser"[tiab] OR "thulium fibre laser"[tiab] OR "TFL"[tiab] OR "thulium laser"[tiab]) AND ("holmium:yag"[tiab] OR "holmium yag"[tiab] OR "holmium laser"[tiab] OR "Ho:YAG"[tiab]) AND ("pulse modulation"[tiab] OR "pulse-modulated"[tiab] OR "modulated pulse"[tiab] OR "Moses"[tiab] OR "Virtual Basket"[tiab] OR "Vapor Tunnel"[tiab] OR "Moses 2.0"[tiab]) AND (lithotripsy[tiab] OR "laser lithotripsy"[tiab] OR ureteroscopy[tiab] OR "retrograde intrarenal surgery"[tiab] OR "RIRS"[tiab] OR "ureterorenoscopy"[tiab] OR urolithiasis[tiab] OR "kidney stone"[tiab] OR "renal stone"[tiab]))	PubMed / PMC / Medline	43
("thulium fiber laser" OR "thulium fibre laser" OR TFL OR "thulium laser") AND ("holmium yag" OR "holmium:yag" OR "holmium laser" OR Ho:YAG) AND ("pulse modulation" OR "pulse-modulated" OR "Moses" OR "Virtual Basket" OR "Vapor Tunnel" OR "Moses 2.0") AND (lithotripsy OR "laser lithotripsy" OR ureteroscopy OR "retrograde intrarenal surgery" OR RIRS OR ureterorenoscopy OR urolithiasis OR "kidney stone" OR "renal stone")	Cochrane Library	24
1. 'thulium fiber laser':ab,ti OR 'thulium fibre laser':ab,ti OR TFL:ab,ti OR 'thulium laser':ab,ti 2. 'holmium yag':ab,ti OR 'holmium:yag':ab,ti OR 'holmium laser':ab,ti OR 'Ho:YAG':ab,ti 3. 'pulse modulation':ab,ti OR 'pulse-modulated':ab,ti OR 'Moses':ab,ti OR 'Virtual Basket':ab,ti OR 'Vapor Tunnel':ab,ti OR 'Moses 2.0':ab,ti 4. 'lithotripsy'/exp OR 'ureteroscopy'/exp OR 'retrograde intrarenal surgery'/exp OR 'urolithiasis'/exp OR 'kidney stone'/exp OR 'renal stone'/exp 5. 1 AND 2 AND 3 AND 4 6. Limit 5 to human AND English language AND yr="2017 -Current"	Embase (via Ovid)	24
